# Barriers and facilitators associated with the upscaling of the Transmural Trauma Care Model: a qualitative study

**DOI:** 10.1186/s12913-024-10643-7

**Published:** 2024-02-13

**Authors:** Julia Ratter, Suzanne Wiertsema, Ilham Ettahiri, Robin Mulder, Anne Grootjes, Julia Kee, Marianne Donker, Edwin Geleijn, Vincent de Groot, Raymond W. J. G. Ostelo, Frank W. Bloemers, Johanna M. van Dongen

**Affiliations:** 1 Amsterdam UMC, location AMC, Department of Rehabilitation Medicine, Amsterdam Movement Sciences, Meibergdreef 9, Amsterdam, The Netherlands; 2 Amsterdam UMC, location AMC, Department of Trauma Surgery, Amsterdam Movement Sciences, Meibergdreef 9, Amsterdam, The Netherlands; 3 Amsterdam UMC, location VUmc, Department of Rehabilitation Medicine, Amsterdam Movement Sciences, De Boelelaan 1117, Amsterdam, The Netherlands; 4 Department of Health Sciences, Faculty of Science, Vrije Universiteit Amsterdam, Amsterdam Movement Sciences, Amsterdam, The Netherlands; 5 Amsterdam UMC, Department of Epidemiology and Data Science, location VUmc, Amsterdam Movement Sciences, De Boelelaan 1117, Amsterdam, The Netherlands

**Keywords:** Trauma, Fractures, Framework method, Constellation approach, Implementation, Process evaluation, Rehabilitation

## Abstract

**Background:**

To assess the barriers and facilitators associated with upscaling the Transmural Trauma Care Model (TTCM), a multidisciplinary and patient‐centred transmural rehabilitation care model.

**Methods:**

Semi-structured interviews were conducted with eight trauma surgeons, eight hospital-based physiotherapists, eight trauma patients, and eight primary care physiotherapists who were part of a trauma rehabilitation network. Audio recordings of the interviews were made and transcribed verbatim. Data were analysed using a framework method based on the “constellation approach”. Identified barriers and facilitators were grouped into categories related to structure, culture, and practice.

**Results:**

Various barriers and facilitators to upscaling were identified. Under structure, barriers and facilitators belonged to one of five themes: “financial structure”, “communication structure”, “physical structures and resources”, “rules and regulations”, and “organisation of the network”. Under culture, the five themes were “commitment”, “job satisfaction”, “acting as a team”, “quality and efficiency of care”, and “patients’ experience”. Under practice, the two themes were “practical issues at the outpatient clinic” and “knowledge gained”.

**Conclusion:**

The success of upscaling the TTCM differed across hospitals and settings. The most important prerequisites for successfully upscaling the TTCM were adequate financial support and presence of “key actors” within an organisation who felt a sense of urgency for change and/or expected the intervention to increase their job satisfaction.

**Trial registration:**

NL8163 The Netherlands National Trial Register, date of registration 16-11-2019.

**Supplementary Information:**

The online version contains supplementary material available at 10.1186/s12913-024-10643-7.

## Background

Major trauma is one of the leading causes of death and disability [1, 2]. Typically, trauma patients are relatively young and the sustained injuries not only adversely affect health and wellbeing [3], but also result in a high number of disability-adjusted life years (DALYs) [4–6]. In addition to the human impact of traumatic injuries, their economic impact can also be substantial [7]. For example, an estimate of the total societal cost of traumatic injuries in the Netherlands in 2017 was €3.5 billion [8, 9]. Increased levels of absenteeism and lost productivity while being at work (i.e., presenteeism) account for the majority of these costs [10].

In recent decades, the optimisation of pre- and in-hospital trauma care has led to a notable decline in trauma-related morality rates and evolved to such an extent that further reductions in mortality are expected to be marginal [11]. As such, the focus of both trauma care and research has shifted towards improving the rehabilitation process [2, 12–14]. To illustrate, Brooke et al. [15] compared the effect of early consultation with a rehabilitation physician and pain management, physiotherapy, psychological treatment, and further specialist referrals (i.e., early rehabilitation intervention) with usual care in patients who were in motor vehicle accidents. The findings showed that early rehabilitation intervention resulted in significant improvements in pain and earlier return to previous activities. Bouman et al. [16] investigated the effect of coordinated care by a trauma surgeon and a rehabilitation physician (i.e. so-called fast-track rehabilitation) for patients with multiple trauma. The results showed that fast-track rehabilitation led to faster recovery in functional status during six months of follow-up.

To improve the rehabilitation process of patients with traumatic injuries in the Netherlands, the Transmural Trauma Care Model (TTCM) was developed. The TTCM consists of the following four features: 1) A joint outpatient consultation with a trauma surgeon and a hospital-based physiotherapist (HBP); 2) Rehabilitation care provided by a physiotherapist belonging to network of specialised primary/tertiary care trauma physiotherapists (referred to as network physiotherapist [NPs] in the Dutch setting); 3) Continuous alignment of treatment goals between the multidisciplinary hospital team and specialised NPs, and 4) Encrypted and continuous email contact between HBPs and NPs throughout the patients’ rehabilitation process.

A pilot study showed that implementing the TTCM in a Dutch Level-1 trauma centre was feasible, had the potential to improve patient outcomes and patient satisfaction, and may reduce costs [17, 18]. However, two key challenges were ensuring that information sharing between primary care (e.g., general practitioners and physiotherapy practices) and secondary care (e.g., hospital-based care services) providers was consistent and timely, and funding for the HBPs was arranged. Based on these findings, the original TTCM was updated and recently implemented in a larger number of hospitals with the aim of evaluating TTCM’s effectiveness and cost-effectiveness [19]. This process of expanding and replicating an innovative pilot project in more and different hospitals is known as “upscaling”, and is a complex process that depends heavily on context [20–23]. Currently, it is not known if the TTCM can be implemented successfully in Dutch hospitals that were not involved in its initial development. Therefore, this study aims to assess the barriers and facilitators associated with successful upscaling TTCM in the Netherlands.

## Methods

### Study design and setting

This study was conducted alongside a multicentre trial that aims to evaluate the effectiveness and cost-effectiveness of TTCM in nine Dutch hospitals [19]. The research team at AmsterdamUMC, location VUmc, coordinated and supervised the implementation of the TTCM at each site. The implementation process involved using procedures tailored to each hospital’s respective context [24, 25]. The methods for conducting the current process evaluation were based on those described in Wiertsema et al. [18] and the guideline for evaluating implementations in healthcare [26]. The study was reported according to the COnsolidated criteria for REporting Qualitative research (COREQ) checklist [27] (supplementary file 1).

### Participant recruitment

Participants were purposively selected from the nine hospitals involved in the aforementioned multicentre trial. The relevant stakeholders that were represented included trauma surgeons, NPs, HBPs, and patients. Three researchers (JR/SW/JvD) were responsible to recruiting participants. The recruitment procedure involved contacting potential participants via email or telephone, explaining the study purpose and procedures, and inviting them to participate in the study. Care was taken to include healthcare providers and patients who were positive about the TTCM as well as those who were not. If potential participants were willing to participate and gave informed consent, an in-person interview was scheduled at a time and location convenient to the participants. An interview by video conferencing was also an option.

### Data collection

Data were collected using semi-structed interviews. These interviews were conducted by a two- or three-person team, consisting of a (3rd-year) student enrolled in a Bachelor of Health Sciences degree program at the VU University and one or two researchers (JR/RO/JvD/SW). The professional and academic backgrounds of the researchers were as follows: clinical epidemiology (JR/RO), human movement sciences (JvD/SW), physiotherapy (JR/SW/RO), or health technology assessment (JvD). Two researchers (JvD/SW) were experienced in conducting qualitative research [19, 28, 29] and all four student interviewers had successfully completed coursework on qualitative research and interviewing methods. Before the formal interviews were conducted, all interview team members were trained on procedures.

In sum, interviews were guided by a topic list and an audio recording was made [30]. Topic lists were based on the literature, a theoretical framework (see section on data analysis), and previous experience [18]. During the interview phase of the study, the topic list was adjusted based on knowledge and experience from previous interviews and adapted to the stakeholder in question [28] (supplementary file 2). The interview procedure involved one researcher leading the interview, while the other(s) probed areas for further questioning, kept track of the topic list, and made notes. Researcher objectivity was optimised by keeping a reflective diary [29]. To enhance the data’s trustworthiness, a member check was performed after each interview by sending the participants a brief summary of the interview and its transcript [31]. The interviews were conducted between April 2022 and August 2022.

### Data analysis

Descriptive statistics were used to analyse participant characteristics (i.e., age, gender, stakeholder, and if applicable, years of professional experience, experience with TTCM [yes/no], type of injury, and time since discharge) and the degree to which certain parts of the TTCM were implemented/upscaled (i.e., reimbursement of the HBP, joint outpatient consultations, the exchange of patient information, accreditation of the network). For this, the following variables were described and compared between university medical centres and supra-regional hospitals: reimbursement of the HBPs (i.e., completely, partially, or not), care providers acted as a team (i.e., completely, partially, or no), information exchange between primary and secondary care (i.e., yes/no), and accreditation (i.e., was arranged for the network activities, yes/no).

**Fig. 1:**
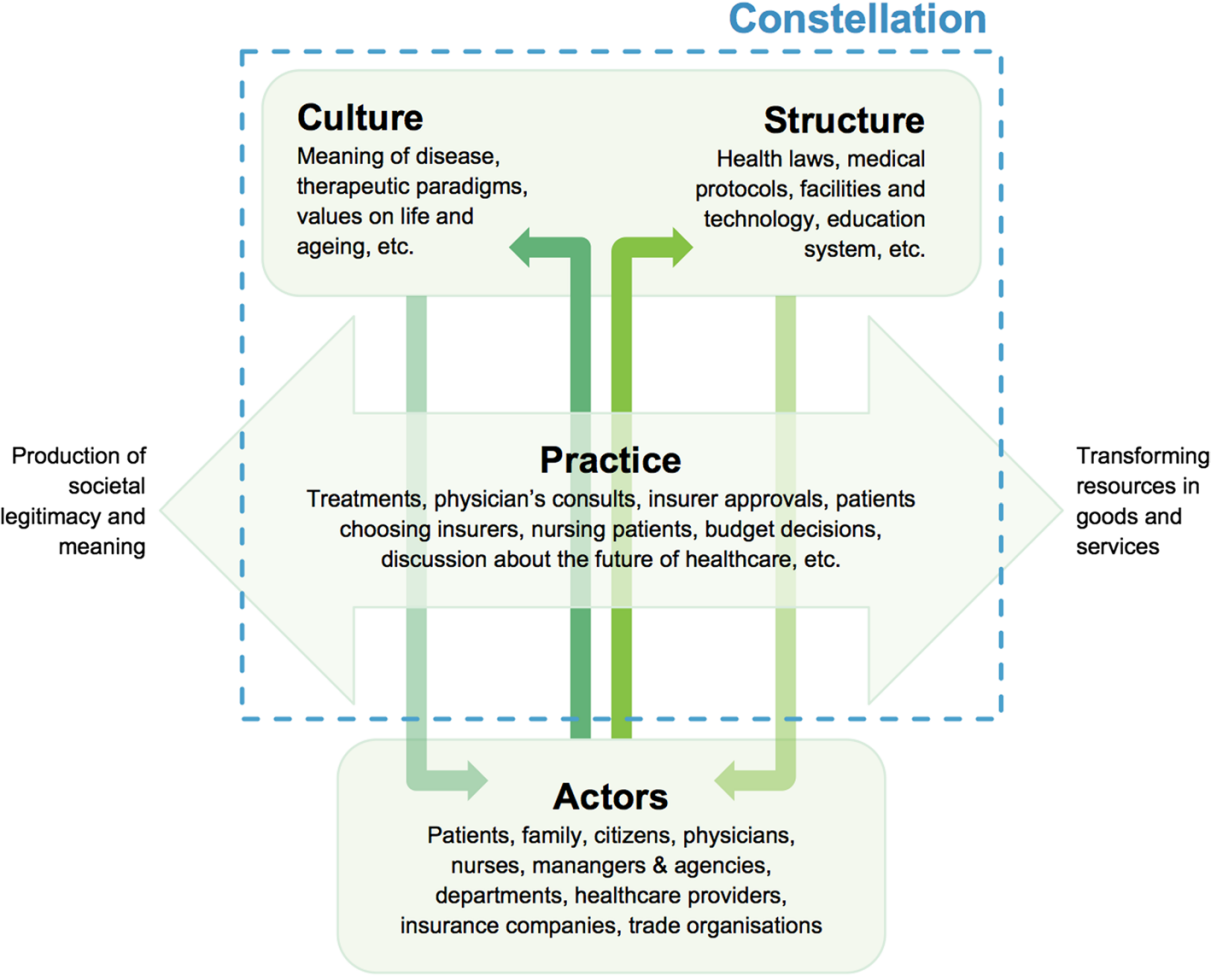
The interaction between the three elements of the ‘structure culture and practice triplet’ within a constellation.

Data from the interviews were analysed using a framework method, a hierarchical, matrix-based method for ordering and synthesising qualitative data [32]. Our theoretical framework was based on the “constellation approach”, which assumes that a healthcare system consists a set of interrelated practices and relevant, interrelated, structuring elements that define and fulfill a function in the more extensive system as in a constellation [22]. Within a constellation, there is a continuous interaction between the “structure, culture and practice triplet” (Fig. 1). A more detailed description of the constellation approach can be found in Supplementary file 3.

The applied framework method consists of seven steps. First, we transcribed the recorded interviews verbatim (IE/RM/AG/JK). Second, we familiarised ourselves with the content in the interviews by listening to the audio recordings and rereading the transcripts (IE/RM/AG/JK/JR). Third, we labelled text fragments relevant to the research question by relevant codes (open coding)(IE/RM/AG/JK and JR). Fourth, we developed a working analytical framework by grouping codes according to structure, culture, and practice categories of the constellation approach (IE/RM/AG/JK/JR). We developed final codes by applying an iterative process of refining through discussion until the criterion of saturation (i.e., no novel codes emerged from subsequent iterations) was met (JR/RO/JvD). Our approach to identifying themes and codes was both deductive and inductive: we used themes and codes defined by Wiertsema et al. [18] as a starting point (deductive), while new themes and codes were generated from the data (inductive). Fifth, working in pairs, we systematically reread each transcript, highlighted each meaningful text passage, and selected and attached an appropriate code from the final analytical framework (IE/RM/AG/JK/JR/JvD). Sixth, we charted the data by generating a framework matrix in which data were summarised by category and stakeholder group, categorised into the matrix, followed by adding illustrative quotes from participants to the matrix (IE/RM/AG/JK/JR). Lastly, we used the framework matrix to interpret the data together with the interview/coding notes. Two researchers (JR/JvD) assessed the “value” of the participants’ statements based on the intensity, frequency, persuasiveness, and contrast with which they were made. To ensure rigour and credibility, two other researchers (SW/RO) reviewed the generated matrix and checked whether the selected quotes were relevant to the themes. Disagreements were resolved by discussion. All steps were conducted using word-processing software. Quotes were translated from Dutch to English by an English native speaker and were edited slightly to make them more readable without losing their meaning.

## Results

### Participants and setting

A total of 33 stakeholders were invited to participate; however, one trauma surgeon declined the invitation due to limited availability. In the end, 32 interviews (31 via Zoom/TEAMS; one in-person) were conducted with eight trauma surgeons, eight NPs, eight HBPs, and eight patients. Five (63%) of the trauma surgeons, seven (88%) NPs, and four (50%) HBPs worked at a university medical centre. Six patients (75%) were treated at a university medical centre. The healthcare providers’ professional experience ranged from 2 to 40 years (mean=11.78; SD=10.09) and their experience with the TTCM ranged from 1 to 54 months (mean=15.19; SD=11.13)(Table 1).

**Table 1:** Characteristics participants

Gender	Age (years)	Professional experience (month)	Experience TTCM (month)	Affiliation with one of the participating University Medical Centers or supra-regional hospitals	Stakeholder	Kind of injury	Time since discharge (month)
female	26	0- 5	20-25	supra-regional hospital	HBP	not applicable	not applicable
male	30	0- 5	20-25	University Medical Center	HBP	not applicable	not applicable
female	41	10-15	30	supra-regional hospital	HBP	not applicable	not applicable
male	25	0- 5	10-15	University Medical Center	HBP	not applicable	not applicable
male	63	40-45	5-10	University Medical Center	HBP	not applicable	not applicable
female	29	5-10	15-20	supra-regional hospital	HBP	not applicable	not applicable
female	45	20-25	15-20	University Medical Center	HBP	not applicable	not applicable
female	25	0- 5	30-35	supra-regional hospital	HBP	not applicable	not applicable
male	47	10-15	5-10	supra-regional hospital	trauma surgeon	not applicable	not applicable
male	53	15-20	15-20	University Medical Center	trauma surgeon	not applicable	not applicable
male	37	0- 5	15-20	University Medical Center	trauma surgeon	not applicable	not applicable
male	38	5-10	5-10	supra-regional hospital	trauma surgeon	not applicable	not applicable
male	38	5-10	15-20	University Medical Center	trauma surgeon	not applicable	not applicable
male	50	10-15	5-10	University Medical Center	trauma surgeon	not applicable	not applicable
male	39	5-10	0-5	supra-regional hospital	trauma surgeon	not applicable	not applicable
male	39	0- 5	15-20	University Medical Center	trauma surgeon	not applicable	not applicable
male	65	not applicable	not applicable	University Medical Center	patient	Collarbone fracture and torn tendons by making a rollover	0-5
female	55	not applicable	not applicable	supra-regional hospital	patient	Both wrists fractured, both sides radius and ulna fractured by slipping on a frozen puddle	5-10
female	60	not applicable	not applicable	University Medical Center	patient	Fractured shoulder/upper arm due to slipping	0-5
male	54	not applicable	not applicable	University Medical Center	patient	Fractured left fibula and right shoulder	0-5
male	59	not applicable	not applicable	University Medical Center	patient	Fall with fracture of the back part of the foot	5-10
male	48	not applicable	not applicable	supra-regional hospital	patient	Right shoulder fracture due to skiing at low speed	0-5
male	48	not applicable	not applicable	University Medical Center	patient	Six broken ribs, a broken wrist, a broken hip and a broken tibia plateau due to a bike accident	5-10
male	67	not applicable	not applicable	University Medical Center	patient	Broken little finger due to black out and fall on a grate	0-5
male	50	25-30	10-15	supra-regional hospital	NP	not applicable	not applicable
male	38	10-15	5-10	University Medical Center	NP	not applicable	not applicable
male	35	5-10	10-15	supra-regional hospital	NP	not applicable	not applicable
male	50	25-30	50-55	University Medical Center	NP	not applicable	not applicable
male	29	5-10	10-15	University Medical Center	NP	not applicable	not applicable
male	54	30-35	10-15	supra-regional hospital	NP	not applicable	not applicable
female	31	5-10	10-15	University Medical Center	NP	not applicable	not applicable
female	49	10-15	5-10	supra-regional hospital	NP	not applicable	not applicable

Of the participating hospitals, one had successfully arranged reimbursement for the HBP at the outpatient trauma clinic, three had partially arranged it, and five had not made any arrangements for reimbursement. Additional findings on the extent to which the TTCM was implemented are summarised in Table 2.

**Table 2:** Degree of implementation

Participating hospital and affiliated networks	Completely reimbursement of the HBP at the outpatient trauma clinic	Partially reimbursement of the HBP at the outpatient trauma clinic	No reimbursement of the HBP at the outpatient trauma clinic	Completely ‘Acting as a team’ at the outpatient clinic for trauma patients’	Partially ‘Acting as a team’ at the outpatient clinic for trauma patients’	No ‘Acting as a team’ at the outpatient clinic for trauma patients’	Exchange of patient information/data between the networks and hospitals (and vice versa) arranged	Accreditation of network activities is arranged
University Medical Centers	0/4	2/4	2/4	1/4	1/4	2/4	2/4	1/4
Supra‐regional hospitals	1/5	1/5	3/5	0/5	2/5	3/5	2/5	2/5

### Barriers and facilitators

Stakeholders shared the belief that the TTCM held the potential to improve both the quality and efficiency of trauma rehabilitation. Nonetheless, various barriers and facilitators associated with the upscaling of the TTCM were identified for each category of the constellation approach and are discussed below. Similarities and differences between the various stakeholders also were observed. An overview of all themes, sub-themes, and illustrative quotes are presented in Table 3.

**Table 3:** Facilitators (F) and barriers (B) expressed by care-providers and patients regarding the implementation of the TTCM, related to structure, culture and practice. Quotes are from trauma patients (P), trauma surgeons (T), hospital-based physiotherapists (HBP), and network physiotherapists (NP)

**Level**	**Theme**	**Subtheme**	**Facilitator**	**Barrier**	**Stakeholders**	**Illustrative quote**
**Structure**	Communication structure	Secured email system	Use of a secured email system between hospitals and network practices	Electronic patient records in hospitals and network practices are often incompatible	• Trauma surgeons • Hospital‐based physiotherapists • Network physiotherapists	B: ‘The problem is the Electronic Public Records. They aren’t communicating with each other. That’s where the problem lies.’ (R17, NP)
						B: ‘I’m not sure why exactly, but I think my emails from ZorgDomein are not being received.’ (R24, NP)
						F: ‘It’s of course ideal for the network that you can simply send it digitally and securely.’ (R13, HBP)
			Use of a standardized template for the secured email	Standardized template is not implemented in the appropriate software	• Hospital‐based physiotherapists • Network physiotherapists	B: ‘There are still some difficulties. Initially, communication was supposed to be via e-mail, but there still seem to be some issues. I believe it’s being run by the hospital, but the system isn’t fully functional yet.’ (R19, NP)
						F: ‘It’s a standardized list that we fill in, which is very nice because it’s faster and easier. We don't have to rush through things. In principle, you can send it in straight away. The process costs you very little time.’ (R12,HBP)
				(Changes in) dates and times of the patients’ outpatient appointments are not automatically communicated to the NP/HBP	• Network physiotherapists	B: ‘It does cause some stress or [extra] work because we have to keep track of the patients’ outpatient arrangements or when they need to go for a checkup. And then you still have to write a transfer report.’ (R23, NP)
	Financial structure	Reimbursement	Reimbursement for the HBP at the outpatient clinic for trauma patients has been arranged	Reimbursement for the HBP at the outpatient clinic for trauma patients has not been arranged	• Trauma surgeons • Hospital‐based physiotherapists	B: ‘Look, one has to pay. It’s all about budgeting and whether the department says it won’t reimburse or pay for it. It has nothing to do with a lack of space. I think it's really a matter of finances.’ (R5, T)
						F: ‘… things started in November [2021][start implementation phase February 2021], when all the financing had been arranged. That’s when we launched the outpatient clinic.’ (R12, HBP)
	Physical structures and resources	Availability of rooms and/or computers	Sufficient consultation rooms available	Insufficient consultation rooms available	• Trauma surgeons • Hospital‐based physiotherapists	F: ‘Well, the clinic is so big and has so many rooms available that’s not a problem.’ (R13, HBP)
						B: ‘It would be more helpful if they [HBP] were there with us. Only, in terms of actual rooms, there’s no physical space available. So that’s, of course, a pity. Yes, that’s a problem at our hospital, in my opinion. This must improve if you want to get the most out of it.’ (R4, T)
				Too few computers available	• Hospital‐based physiotherapists	B: ‘We can't type at the same time as the doctor because they’re often behind the computer. So, we often have to do that on the side (after the consultation). This can be quite time-consuming.’ (R14, HBP)
	Rules and regulations		Patients are free to choose their care providers	The lack of guarantee on a high number of referrals for the network physiotherapists	• Trauma surgeons • Network physiotherapists • Patients	B: ‘But we ended up training so many therapists in [name of city]. But if you compare the number of patients per trained therapist in [name of city], I think it's [referrals] very little.’ (R21, NP)
						F: ‘No, just unrestrained. I didn't at all feel controlled or anything.’ (R32, P)
						F: ‘… the patient can choose a physiotherapist himself.’ (R4, T)
				Benchmark (a regulatory tool) limits the number of treatments	Network physiotherapists	B: ‘Yes, in some cases I’d prefer to see certain people three times a week. But I don't because my treatment index limits what I can offer.’ (R18, NP)
				Reimbursement through the basic insurance package is limited	• Network physiotherapists	B: ‘One thing that’s sometimes inconvenient is that the health insurance policy only covers treatment lasting half a year when there are cases when you really do need more time.’ (R24, NP)
						B: ‘If you don’t have supplementary insurance, you’ll have to pay for the first twenty treatments. And if the initial phase includes treatments at home, then this can run up to around 40 euros per treatment. So, after the €285 deductible excess, you’d have to pay an additional €800, more or less. For some people, that’s a lot of money. Occasionally people say, well, I'll use my supplementary insurance, and I'll see what happens. That also happens. And of course this, well… sometimes this, unfortunately, has an impact on the initial goal or the recovery process.’ (R18, NP)
	Organization of the network	Accreditation		Accreditation of the network activities has not been arranged	• Network physiotherapists	B: ‘The meetings have yet to be accredited, so at the moment, it’s completely voluntarily.’ (R21, NP)
		Training and education	Being part of the network is free of charge	Training for the NPs is a prerequisite for joining the network and costs money (e.g. because they had to close the practice)	• Network physiotherapists	B: ‘The only investment we had to make is to take a course… [I] watched some presentations by trauma surgeons. I think it cost more than six hundred euros. And the annoying thing was, this wasn’t possible during the weekend, so I also had to take three days off. So I also lost three days’ revenue because I had to close [my practice] for three days.’ (R17, NP)
						F: ‘No, no, the only investment we had to make is that we had to take a course.’ (R17, NP)
			The fact that the training was online due to the Covid-19 pandemic made participating more feasible from a logistical perspective	The fact that the training was online due to the Covid-19 pandemic resulted in fewer possibilities for personal interaction	• Network physiotherapists	F/B: ‘Everything took place online, so it was all a bit detached and impersonal during this Corona time. I think this was a plus, especially considering the travel time to the north of the country. But if you want more interaction, I think one should arrange live or face-to-face meetings.’ (R18, NP)
			The duration of the training was good	The training could have been shorter	• Network physiotherapists	B: ‘As far as I am concerned, the training could easily have been completed in two days.’ (R17, NP)
						F: ‘Yes, I thought the duration [of the course] was okay. I think three days was in itself good as a basis.’ (R18, NP)
			Content of the training was of added value for the treatment of patients with trauma	Training lacked some topics/content relevant to the treatment of trauma patients	• Network physiotherapists	B: ‘Maybe the psychological aspect of the trauma process could also be looked at a little more. This doesn’t always receive the same amount of attention.’ (R20, NP)
						B: ‘My biggest problem was that I found the second training quite bad, to be honest. This was because there was a lot of overlap between what the various doctors said.’ (R22, NP)
						F: ‘Yes, to some degree. You learn to look more critically at things, especially at the burden you may be placing on your patients. So in this sense, certainly.’ (R17, NP)
		Website	Having an appropriate and up-to-date website	More information about the NPs (e.g. expertise) on the website would be useful	• Patients	B: ‘It would be useful to provide a description of the specialties of the physiotherapists on the list that’s handed out.’(R25, P)
**Culture**	Commitment	Commitment at the hospital	High intrinsic motivation of TTCM teams and colleagues of other relevant departments (e.g.: trauma surgery, rehabilitation medicine)	Low intrinsic motivation of TTCM teams and colleagues of other relevant departments (eg: traumasurgery, rehabilitation medicine)	• Hospital‐based physiotherapists	B: ‘If they can tackle it at the front-end, so to speak so that also my colleagues [could be involved] and not just me. This way, it will also ‘come to life’ more, also in the department. I think this is one area where we could improve.’ (R10, HBP)
						B: ‘We have a surgeon who is very enthusiastic about it. But he communicated very little about it with other surgeons [from other departments].’ (R9, HBP)
						B: ‘… it’s a logistical issue involving your work, so to speak. You have to take, well, your colleagues have to grant you the space to take the time you need to be there.’ (R11, HBP)
						F: ‘I’ve noticed that if you build something together from scratch [network], you’re inclined to make sure it’s a success; maybe just take those extra steps, call again, or send an e-mail or describe things in more detail...’ (R10, HBP)
		Commitment at the network	High intrinsic motivation of the participating network physiotherapists to be part of a network	Low intrinsic motivation of the participating network physiotherapists to be part of a network	• Network physiotherapists	B: ‘Honestly, I feel I need to say that… well, many people currently involved in the trauma network didn’t join because they are interested in trauma patients. They seem more interested in just being part of a network.’ (R22, NP)
						F: ‘You have a group of therapists who are motivated to do something with it. Otherwise, you wouldn’t follow the training.’ (R21, NP)
	Acting as a team	Contact trauma surgeons and hospital‐based physiotherapists	Care-providers at the outpatient clinic act as a team during the joint consultations	Care-providers work separately from each other during the outpatient consultations	• Trauma surgeons • Hospital‐based physiotherapists • Patients	B: ‘So, three times people [the trauma surgeon and the HBP] came by, and I just sat there alone in that room, and that felt strange... […] they each came by and sent the other over, but they never visited me at the same time. Well, I just sat in that chair and wondered: ‘What now?’’ (R26, P)
						B: ‘We don't have fixed days when we’re present at the trauma clinic because the surgeons don't want us in the room with them. So yes, we have a separate room.’ (R9, HBP)
						F: ‘It was a shared effort. The surgeon explained what had taken place and what he had done [during the surgery], and the physiotherapist indicated what I can do. Of course, there was also the treatment by another physiotherapist affiliated with the trauma [network].’ (R29, P)
						F: ‘… Yeah, as colleagues among colleagues, it all ran very smoothly. There just didn’t seem to be any [professional] borders getting in the way.’ (R12, HBP)
						F: ‘And I also like is that you immediately have more eyes, and they looked at them [the trauma patients] with a slightly different view. Look, we do a trick during the operation, then our work is done for the most important part. So in that…. we are also very often dependent on the physiotherapist’s work.’ (R4, T)
			Awareness of responsibilities, leadership, and professional boundaries: care-providers at the outpatient clinic (trauma surgeon and HBP) take pro- fessional boundaries into account		• Trauma surgeons	F: ‘So, there’s this patient where I still have to look at the wound to see if the fracture isn’t healing properly yet, and the physiotherapist then takes a bit of a step back. So it’s clear to us what our responsibilities are.’ (R2, T)
		Contact between network and hospital team	The possibility of low-threshold contact between network and hospital team	Inconsistent feedback loop between network and hospital team	• Trauma surgeons • Hospital‐based physiotherapists • Network physiotherapists • Patients	B: ‘Sometimes, the feedback sent from primary care to us is a bit lacking. And, of course, we [only] see what takes place at the outpatient clinic. So, I don't know how people perform their exercises at home, and I don't always know if what people tell me in the doctor's office is the actual truth. This is why I think feedback is so important. This is where we should establish a smoother or better feedback loop.’ (R3, T)
						F: ‘The advantage of having a physiotherapist is that you can just contact them, and they can then easily contact the trauma surgeon.’ (R20, NP)
						F: ‘It’s easier when the physiotherapist [HBP] is there because they can then take over that task, thus bridging the gap in communication.’ (R1, T)
						B: ‘Only what I understood from my physiotherapist. He had questions regarding certain pains I have at the moment. But these haven’t been answered yet. It’s been two weeks now, and I have no explanation for this yet.’ (R29, P)
						F: ‘The times I was in contact with them were very pleasant. We could just talk to each other, as colleagues. So, I think the cooperation is very balanced.’ (R23, NP)
						F: ‘I think it’s a big plus for patient satisfaction or patient-friendliness.’ (R4, T)
	Quality and efficiency of care	Contact between care providers and patients	Care-providers think that the TTCM, which amongst others, improved the level of contact between different care provides, enhances the quality and efficiency of care		• Trauma surgeons • Hospital‐based physiotherapists • Network physiotherapists	F: ‘I think that good collaboration between primary and secondary care can reduce that kind of risk, improve cooperation, and also improve the speed and agility of care.’ (R21, NP)
						F: ‘You are able to provide more efficient outpatient services.’ (R2, T)
						F: ‘[Yes, excellent, very nice.] I think good cooperation between primary and second care [the TTCM] is greatly lacking within the Dutch healthcare system.’(R21, NP)
		Workload	Lower administrative workload for trauma surgeon		• Trauma surgeons	F: ‘I think the administrative workload has decreased, even for surgeons.’ (R2, T)
		Applicability of the TTCM	Presence of a hospital-based physiotherapist at the joint consultations is particularly useful – and of added value - for complex injuries	The HBP does not have an added value at the joint consultations for every patient	• Trauma surgeons • Hospital‐based physiotherapists	B: ‘No, [multidisciplinary collaboration] is not always an equally useful contribution for [trauma patients].’ (R4, T)
						F: ‘For certain patient categories, yes, especially regarding more complex cases, such as patients receiving multidisciplinary treatment or patients with multiple injuries. I think that's the most important thing.’ (R8, T)
						F: ‘Yes, I just think it [the TTCM] should happen nationwide, especially in large trauma centers with patients dealing with multiple trauma.’ (R5, T)
	Patients experience		Patients receive a clear treatment plan	Patient is not aware of what has been communicated between hospital‐based physiotherapists and network physiotherapists	• Patients	F: ‘I do know that my physiotherapist [network physiotherapist] sent feedback to the hospital's physiotherapist before the final interview with the doctor.’ (R30, P)
			Patients feel heard	Care providers sometimes contradict each other	• Hospital‐based physiotherapists • Patients	B: ‘Doctors and physiotherapists don't go well together. That’s often the old practice, especially in the Netherlands. While it's precisely the combination of recovery and [a focus on] the body – actually moving and building things up again – that can help.’ (R30, P)
						B: ‘So, the physiotherapist who released me from the hospital gave me a schedule with exercises. But when I eventually had a consultation with the hand physiotherapist, they never referred to those exercises at all. They gave me completely different exercises, from which I benefitted much more.’ (R26, P)
						F: ‘No, no, they certainly heard me, and it was an empathetic conversation. I wasn’t sent away by anyone, and I was given the time I needed. So I didn't get kicked out, no.’ (R25, P)
						F: ‘Now that I've been through this whole process, I have to say that my confidence in the Dutch healthcare system has increased. Not that I had little faith in it, but I eventually felt supported by the fact that all this [collaboration between care providers] is possible. Yeah, so it gave me courage.’ (R26, P)
			Patients are satisfied with the care that they received	Patients feel like the care process was somewhat rushed	• Patients	B: ‘I had the feeling that things were being rushed, that it was just hectic. I understand that, I'm busy too sometimes. But, well…’ (R26, P)
						F: ‘I do have the feeling that going to the physiotherapist at a relatively early stage ensured that my shoulder soon regained freedom [of movement], and I suffered less in the long term.’ (R30, P)
						F: ‘Yes, I liked the joint consultation with the physiotherapist and doctor, trauma doctor, or surgeon. Also because they could respond to each other, which they did. So when one of them said something, another immediately gave an answer, which gave clarity to what, why, and how. So yes, it was very clear.’ (R27, P)
	Job satisfaction		Care providers indicated that they were more satisfied with their job after the implementation of the TTCM		• Trauma surgeons • Hospital‐based physiotherapists • Network physiotherapists	F: ‘Well, at [hospital], I'm just super happy with how things are going. And I’m also very satisfied with the meetings.’ (R24, NP)
						F: ‘So yes, they are just two different specializations present in one place at the same time. And I’m personally very excited about this.’ (R3, T)
						F: ‘So it's, well, actually, I'd rather spend my full twenty-six hour working week just working with TTCM.’ (R11, HBP)
						F: ‘So that just makes it a really fun group. Let me put it this way: it makes it all just a little more satisfying.’ (R20, NP)
**Practice**	Practical issues at the outpatient clinic		Sufficient consultation rooms available	Insufficient consultation rooms available	• Trauma surgeons • Hospital‐based physiotherapists	B: ‘It would be more helpful if they [HBP] were there with us. Only, in terms of actual rooms, there’s no physical space available. So that’s of course, a pity. Yes, that’s a problem at our hospital, in my opinion. This must improve if you want to get the most out of it.’ (R4, T)
						B: ‘...if there are any additional questions, or if I think, well, I’d actually like to spend some more time with them, I don't currently have the time or space for that as things are.’ (R16, HBP)
						F: ‘Well, the outpatient clinic is so big and has so many rooms available that’s not really a problem.’ (R13, HBP)
				Too few computers available	• Hospital‐based physiotherapists	B: ‘We can't type at the same time as the doctor because they’re often behind the computer. So, we often have to do that on the side (after the consultation). This can be quite time-consuming.’ (R14, HBP)
	Knowledge gained	Knowledge exchange between care providers	Trauma surgeons and hospital-based physiotherapists at outpatient clinic learn from each other’s field/profession		• Network physiotherapists • Trauma surgeons • Hospital‐based physiotherapists	F: ‘Of course, we bring along the know-how of the injury and exactly what kind of surgery we’ve performed (if we operated). So that is our know-how. But they really take care of the movement issues and really know how the physiotherapist works in the network practice. [So, they can do that, yes, they can do that too, they may be talking at that level]. I think that's an advantage.’ (R6, T)
			Network physiotherapists gain knowledge and expertise in trauma rehabilitation		• Trauma surgeons	F: ‘Insight into the various fracture treatments has improved, and this knowledge actually expands via the network.’ (R2, T)
			Network physiotherapists gain knowledge and expertise in trauma rehabilitation		• Trauma surgeons	F: ‘Well, through the trauma network, you can refer more directly to physiotherapists who are involved with trauma patients. So it’s no longer enough to send a patient with an ankle fracture to go to the nearest general physiotherapist, who may have never or only incidentally dealt with trauma patients and doesn’t know what to do.’ (R1, T)

### Structure category

With regards to the structure category, five themes were identified: “communication structure”, “financial structure”, “physical structures and resources”, “rules and regulations”, and “organisation of the network”. Each theme was associated with its unique barriers and/or facilitators.

"Communication structure" refers to the exchange of patient information between primary and secondary care. Typically, this takes place via an encrypted email system (i.e., ZorgMail) that allows healthcare providers to send and receive messages, documents, and images securely. The use of this system was perceived as both a facilitator and a barrier. On one hand, communication was sometimes hampered by incompatibility between a given hospital’s electronic patient record system and that of a network or a primary care practice, resulting in extra work (i.e., healthcare providers had to write separate emails instead of the information being automatically transferred). One NP noted:

*‘The problem is the Electronic Patient Record System. They aren’t communicating with each other.’*(R17,NP)

Some HBPs considered alternative encrypted email systems (e.g., ZorgDomein), but these systems had similar incompatibility issues. If, however, the electronic patient record system and the encrypted email system were compatible, the communication structure was perceived as a facilitator.

"Financial structure" refers to the reimbursement of HBPs. The lack thereof was deemed an critical barrier to implementing TTCM by all healthcare providers. One trauma surgeon noted:

*‘…. It’s all to do with budgeting and whether the department says it won’t reimburse or pay for it. It has nothing to do with a lack of space. I think it's really a matter of finances.’*(R5,T)

In hospitals that were successful in securing full reimbursement for the HBP, HBPs were able to be present during all joint outpatient trauma consultations. In most hospitals, however, only partial reimbursement could be achieved (e.g., as in for a limited proportion of the consultations and/or for a limited period); thus, joint outpatient trauma consultations were performed inconsistently or offered only temporarily.

"Physical structures and resources" refers to the availability of adequate rooms and number of computers to conduct joint consultation. Trauma surgeons and HBPs noted that the implementation of the TTCM was sometimes hampered by a lack of adequate rooms and/or an insufficient number computers. In some hospitals, the problem of insufficient resources was pronounced by the wish of trauma surgeons to work separately from HBPs, and hence requiring two rooms per consultation. In other hospitals, the number and size of rooms was simply insufficient. One trauma surgeon noted, for example, that the presence of a HBP meant that there was no longer space for a medical resident.

"Rules and regulations" refer to existing rules and regulations that impacted the implementation of the TTCM. NPs frequently mentioned that the number of TTCM patients they received was relatively low, because of the freedom to choose, some patients disregarded the referral to a network practice. Additionally, regulatory issues, such as “benchmarking” and “reimbursements” limited the number of physiotherapy sessions per patient. “Benchmarking” refers to the Dutch healthcare performance index that compares the average number of sessions per patient across physiotherapy practices. While the aim of this index is to monitor efficiency, some insurance companies use this index as leverage during contract negotiations with physiotherapy practices and/or audits. For physiotherapy practices, this can translate into less money per session, which in turn negatively impacts treatment decisions [33]. One NP indicated, for example, that even if he/she wanted to treat a certain patient three times a week, he/she would not do so, because of the benchmark. Moreover, the number of physiotherapy sessions that is reimbursed through the Dutch basic insurance package is limited. That is, physiotherapy sessions following a hospital admission are only reimbursed after the 20^th^ session and within the one year following discharge. Even though people have the option to purchase supplemental insurance that would cover physiotherapy sessions prior to the 20^th^ session, only 35% of the Dutch population has this coverage [34].

"The organisation of the network" refers to the set-up, content, website, and accreditation of the network. Physiotherapists were eligible to join a TTCM network after completing an online training on how to provide care according to the TTCM. Due to the COVID-19 pandemic, training sessions were organised online, which was perceived as both a facilitator and a barrier. Most NPs appreciated the convenience of not having to travel for training; however, they also noted a reduction in opportunities for personal interaction and networking. The number and duration of the training sessions differed between networks, and depended on the participating networks’ prior experience.

Most hospitals (*n*=7) had not yet arranged accreditation for their network. NPs perceived this as a barrier, as it rendered the status of their participation in the network as being voluntary. Consequently, when they had to temporarily close their practice to attend network activities, such as training sessions, it resulted in a loss of income. In general, healthcare providers and patients were positive about the TTCM website and believed that up-to-date websites can strengthen a network/intervention. One patient noted, however, that he/she would have liked the website to contain more content about the NPs, such as their expertise.

### Culture category

Five themes were identified: “commitment”, “acting as a team”, “quality and efficiency of care”, “patients’ experience”, and “job satisfaction”.

"Commitment" was the most common theme identified from the interviews and the most contributions came from healthcare providers. This theme refers to their, as well as their colleagues’, intrinsic motivation to work according to the TTCM. A high level of “commitment” was perceived as a facilitator, while a lack thereof was perceived as a barrier. Most healthcare providers were committed and felt some responsibility for the successful implementation of the TTCM. Some HBPs, however, noted that their direct colleagues and/or colleagues from other departments (e.g., trauma surgery) were less committed. In their opinion, this was detrimental to the successful implementation of the TTCM.

"Acting as a team" refers to the “contact between trauma surgeons and hospital-based physiotherapists at the outpatient trauma clinic” and the “contact between the network and the hospital team.” In the hospitals with an inconsistent presence of a HBP during outpatient trauma consultations, both types of contact were affected negatively. In some cases, contact between trauma surgeons and HBPs was limited due to trauma surgeons, contrary to what was intended, expressing the desire to work separately from the HBPs. As one HBP noted:

*‘We don't have fixed days when we’re present at the trauma clinic, because the surgeons don't want us in the room with them. So yes, we have a separate room.’*(R9,HBP)

Some patients also noted that their outpatient consultations were not provided jointly by a trauma surgeon and HBP, which they perceived as a barrier.

Patients and NPs also reported problems with the communication between the hospital team and NPs. As one patient noted:

*‘..what I understood from my physiotherapist… He had questions [for the hospital team] regarding certain pains I have at the moment. But these haven’t been answered yet. It’s been two weeks now and I have no explanation for this as yet.’*(R29,P)

If consultations were provided jointly and an effective communication channel was in place, stakeholders perceived the improved levels of communication between primary care and secondary care as a critical facilitator. When working together, trauma surgeons and HBPs indicated that they were respectful of professional boundaries and that their respective responsibilities were clear. That is, they believed that they complemented each other in terms of knowledge and expertise. Also, most trauma surgeons indicated that their communication with the NPs (via the HBPs) had improved since implementing the TTCM. NPs, on their part, indicated that their contact with the hospital had improved and they believed that they played a more significant role in the rehabilitation process of patients with traumatic injuries.

"Quality and efficiency of care" refers the belief among healthcare providers and patients that the TTCM could enhance the quality and efficiency of trauma care. A trauma surgeon noted:

‘*You are able to provide more efficient outpatient services.*’ (R2, T)

Some healthcare providers indicated, however, that “the applicability of the TTCM” was not always clear. Specifically, they found it challenging to anticipate when and if the presence of a HBP would contribute value to a particular patient's treatment. Trauma surgeons believed that HBPs provided significant added value for patients with complex injuries. In addition, trauma surgeons frequently mentioned that they experienced a “lower administrative workload” since the implementation of the TTCM, because they were no longer responsible for the communicating with NPs.

"Patient experience" refers to the patients’ experience and satisfaction with the TTCM. In some hospitals, patients reported “feeling rushed” or “not feeling heard”. In most of these hospitals, however, HBPs were inconsistently and/or only temporarily present during the outpatient trauma consultations. Some patients also indicated they were unaware of what had been communicated between the hospital and their NP, and/or noticed that “care providers contradicted each other”. As one patient noted:

*‘So, the physiotherapist who released me from the hospital gave me a schedule with exercises. But when I eventually had a consultation with the hand physiotherapist, they never referred to those exercises at all. They gave me completely different exercises, which I benefitted much more from.’*(R26,P)

"Job satisfaction" refers to the anticipated or experienced effect that working according to the TTCM had on the healthcare providers’ job satisfaction after its implementation. One trauma surgeon was particularly enthusiastic about his/her increased collaboration with HBPs:

*‘So yes, they are just two different specialisations present in one place at the same time. And I’m personally very excited about this.’*(R3,T)

A HBP noted that he/she would prefer to spend his/her entire work week treating patients according to the TTCM.

### Practice category

Two themes were identified: “practical issues at the outpatient clinic” and “knowledge gained”.

"Practical issues at the outpatient clinic" refers to the fact that some HBPs and trauma surgeons experienced some practical problems/issues while working with the TTCM. An important practical issue was the lack of appropriate consultation rooms. In some cases, there was a shortage of consultation rooms at their outpatient clinic, which became pronounced when trauma surgeons wanted to work separately from HBPs. In others, there was insufficient space in the available consultation rooms to allow both a HBP and medical resident to be present with the patient, and/or to place enough computers for each healthcare provider to enter notes simultaneously.

‘*We can't type at the same time as the doctor because they’re often behind the computer. So, we often have to do that on the side (after the consultation). This can be quite time-consuming.*’ (R14, HBP)

"Knowledge gained" refers to the fact that most healthcare providers indicated that they gained expertise in treating patients with traumatic injuries since working according to the TTCM. As one trauma surgeon noted:

‘*What you also realise when you share knowledge with each other, is that this increases my insight into how they work, and I think it also affects the physiotherapist’s insight into how we think as surgeons.*’(R2,T)

## Discussion

### Main findings

This study identified various barriers and facilitators associated with the upscaling of the TTCM.

Under the structure category of the “constellation approach”, the main barriers to upscaling the TTCM were “communication structure” (i.e., incompatibility of electronic patient records), “financial structure” (i.e., absence of reimbursement for the HBP), “physical structures and resources” (i.e., unavailability of rooms/computers), “rules and regulations”, and “the organisation of the network” (e.g., online training). Under culture, the presence of “commitment” and “acting as a team during the consultations” were perceived as facilitators and the lack thereof as barriers. In some hospitals, contact between trauma surgeons and HBPs and between the hospital team and NPs was suboptimal and considered a barrier. In hospitals where contact between healthcare providers was improved, the improvement appeared to coincide with two perceived facilitators: increased level of “job satisfaction” and a “lower administrative workload for the trauma surgeons”. Under the practice category, “practical issues at the outpatient clinic” (e.g., inadequate or insufficient consultation rooms) was perceived as a barrier. With regards to “knowledge gained”, most healthcare providers indicated that they appreciated the fact that their expertise in treating patients with traumatic injuries increased since working according to the TTCM. Most stakeholders, including patients, believed that if the barriers were overcome, the TTCM could significantly improve trauma rehabilitation.

### Comparison with the literature and recommendations for practice

In line with the pilot study, we found that most stakeholders, including patients, believed that the TTCM could significantly improve trauma rehabilitation if implemented successfully. Many of the identified barriers and facilitators were in line with those of the pilot study [18]. In both studies, the inability to refer Dutch patients to a designated healthcare provider was identified as a barrier. This interferes with patients with traumatic injuries from receiving treatment from physiotherapists specialised in trauma rehabilitation (i.e. NPs), and impedes effective collaboration between primary and secondary care. Another barrier that was identified in both studies was the challenge stakeholders faced with arranging reimbursement for HBPs. The main reason for this difficulty arises from the entrenched financial boundaries between primary and secondary care in the Netherlands, which have also impeded the reimbursement of various other transmural care models [35, 36]. Bloemen-Vrencken et al. [37], for example, found that organisational and financial constraints interfered with the implementation of a transmural care model for spinal cord injury patients. In the pilot study, efforts to secure funding for the entire TTCM were not successful either, however, full reimbursement for the HBP was arranged by adjusting the pricing of medical specialist care (i.e., the trauma surgeon). We planned on using the same funding strategy in the current multicentre trial, but this was not feasible due to the suspension of negotiations amid the COVID-19 pandemic. This unforeseen circumstance further complicated the intricate challenge of navigating financial and organisational obstacles in the implementation of transmural care. Consequently, outpatient trauma consultations were performed jointly in less than 50% of the participating hospitals, and generally only for a limited proportion of the scheduled consultations and/or a limited period. Another notable discrepancy between the current multicentre trial and the pilot study was the reluctance of certain trauma surgeons to collaboratively conduct outpatient consultations in the present study; this was not the case in the pilot study. This discrepancy is likely explained by the “not-invented-here syndrome”, that is, the tendency of people and organisations to avoid things they did not create themselves [38, 39]. Such an attitude can act as a barrier to upscaling (healthcare) interventions [40]. Indeed, findings from other studies indicate that “key actors”, “ownership”, and “leadership engagement” (i.e., commitment, involvement, and accountability of leaders with the implementation) are conditional requirements for change management, and upscaling activities in particular [35, 36, 40]. Therefore, it is crucial for trauma surgeons, who frequently hold leadership positions in hospitals [41, 42], to serve as "key actors" during the implementation and/or scaling of the TTCM. In an ideal situation, this would be established along with strong support from highly committed HBPs. This might be achieved by providing comprehensive training programs to trauma surgeons, HBPs, and NPs; fostering a culture of collaboration and shared responsibility; and establishing clear communication channels between stakeholders. Furthermore, it is crucial for the overall leadership of a hospital to champion the implementation of a new healthcare intervention, as a supportive organisational environment is a critical success factor for effective implementation and/or upscaling [43, 44]. Another barrier that impacted the upscaling of the TTCM is the fact that many of the electronic patient record systems used in Dutch hospitals are incompatible with the available encrypted email systems. This incompatibility severely complicates communication between primary and secondary care providers, which is an integral part of TTCM and many other transmural care initiatives. Indeed, the challenges of compatibility between electronic patient record systems and encrypted email systems have been identified in a systematic review [45] and emphasises the necessity for standardised communication platforms between primary and secondary care [45–47].

### Strengths and limitations

This process evaluation had several strengths. First, we used a theoretical framework to construct an analytical framework that enabled a systematic exploration of the data. Second, all stakeholders groups who provided treatment according to the TTCM were represented in the study. We made deliberate efforts to include participants from diverse hospitals and networks (including those that were who were positive as well as negative about the TTCM) to enhance the transferability of the results. Third, the credibility of data was improved by performing a member-check [31] and keeping a reflective diary [29]. Finally, to optimise reliability and reproducibility, the role of the researcher, the location, the order of the questions, and the description of the coding were described as precisely as possible [29].

The study also had some limitations. Participants were purposively selected, potentially introducing a bias in the sample towards individuals were more positive about the TTCM than the average healthcare provider or patient. Given that the sample is skewed towards individuals who express higher satisfaction with the TTCM compared to the average healthcare provider or patient, the bias may lead to an overestimation of the observed facilitators. Furthermore, we did not include representatives of other healthcare professionals, such as nurses or orthopedic casting specialists, who might have also been affected by the implementation and/or upscaling of the TTCM. Future research endeavors may benefit from interviewing individuals at different departments to capture a more comprehensive perspective. Furthermore, data were obtained through interviews with researchers involved in the development and/or evaluation of the TTCM, which may have caused “social desirability bias”. Consequently, participants may have overstated their positive experiences with the implementation of or working according to the TTCM. For future research, we therefore recommend researchers to obtain additional data through other methods, such as surveys or focus groups (preferably conducted by researchers who are not involved with the TTCM). Third, it is essential to acknowledge the fact the current study only assessed the barriers and facilitators associated with the upscaling of the TTCM during a period of nine months. However, upscaling procedures in the context of healthcare transitions may unfold over more extended periods [23. As such, we may have missed some barriers and facilitators and/or the identified barriers and facilitators may have been experienced more intensely by the stakeholders due to the fact that implementation process had just started.

## Conclusion

Various barriers and facilitators were found to determine the success of upscaling the TTCM in Dutch hospitals. While many of these barriers and facilitators were similar to those identified in the pilot study, some were notably different. The different findings emphasise that implementation of healthcare interventions and upscaling requires attention to context and the importance of the “not-invented-here syndrome”. The most important prerequisites for successfully upscaling the TTCM were adequate financial support and the presence of “key actors” within an organisation who felt a sense of urgency for change and/or expected the intervention to increase their job satisfaction.

### Supplementary Information

**Additional file 1.**

**Additional file 2.**

**Additional file 3.**

## Data Availability

All data relevant to the study are included in this article or are available as supplementary files.

## Abbreviations

DALYs: disability‐adjusted life years
TTCM: Transmural Trauma Care Model
HBP: hospital-based physiotherapist
NP: network physiotherapist (primary/tertiary care)
Amsterdam UMC: Amsterdam University Medical Center
VUmc: VU medisch centrum
COREQ: COnsolidated criteria for REporting Qualitative research checklist
JR: Julia Ratter
SW: Suzanne Wiertsema
JvD: Johanna van Dongen
IE: Ilham Ettahiri
RM: Robin Mulder
AG: Anne Grootjes
JK: Julia Kee
RO: Raymond Ostelo
SD: Standard Deviation
P: patient
T: trauma surgeon
R: respondent
F: facilitators
B: barriers
COVID-19 pandemic: Coronavirus disease 2019
