# Supplementary material for: Barriers and facilitators associated with the upscaling of the Transmural Trauma Care Model: a qualitative study
Source: BMC Health Serv Res. 2024 Feb 13;24:195. doi: 10.1186/s12913-024-10643-7 (PMC10865621; doi:10.1186/s12913-024-10643-7)
Supplement: Supplementary file 1 — Additional file 1. [file 12913_2024_10643_MOESM1_ESM.pdf]

Consolidated criteria for reporting qualitative studies (COREQ): 32-item checklist

For further information about the COREQ guidelines, please see Tong *et al.*, 2017:
https://doi.org/10.1093/intqhc/mzm042

No.	Item	Description	Section #
**Domain 1: Research team and reflexivity**			
Personal characteristics			
1.	Interviewer/facilitator	Which author/s conducted the interview or focus group?	Methods, Data preparation, page 4
2.	Credentials	What were the researcher's credentials? *E.g. PhD, MD*	Methods, Data preparation, page 4
3.	Occupation	What was their occupation at the time of the study?	Methods, Data preparation, page 4
4.	Gender	Was the researcher male or female?	not stated
5.	Experience and training	What experience or training did the researcher have?	Methods, Data preparation, page 4
Relationship with participants			
6.	Relationship established	Was a relationship established prior to study commencement?	Methods, Data preparation, page 4
7.	Participant knowledge of the interviewer	What did the participants know about the researcher? *E.g. Personal goals, reasons for doing the research*	Relevant information was disseminated in the introduction of the interviewer
8.	Interviewer characteristics	What characteristics were reported about the interviewer/facilitator? *E.g. Bias, assumptions, reasons and interests in the research topic*	Relevant information was disseminated in the introduction of the interviewer
**Domain 2: Study design**			
Theoretical framework			
9.	Methodological orientation and theory	What methodological orientation was stated to underpin the study? *E.g. grounded theory, discourse analysis, ethnography, phenomenology, content analysis*	Methods, Data analysis, page 5, Supplementary file 3
Participant selection			
10.	Sampling	How were participants selected? *E.g. purposive, convenience, consecutive, snowball*	Methods, Data preparation, page 4
11.	Method of approach	How were participants approached? *E.g. face-to-face, telephone, mail, email*	Methods, Data preparation, page 4
12.	Sample size	How many participants were in the study?	Results, page 6
13.	Non-participation	How many people refused to participate or dropped out? What were the reasons for this?	Results, page 6
Setting			
14.	Setting of data collection	Where was the data collected? *E.g. home, clinic, workplace*	Results, page 6
15.	Presence of non-participants	Was anyone else present besides the participants and researchers?	No, but not specifically stated

16.	Description of sample	What are the important characteristics of the sample? *E.g. demographic data, date*	Results, page 6 and Table 1
Data collection			
17.	Interview guide	Were questions, prompts, guides provided by the authors? Was it pilot tested?	Methods, page 4, Supplementary file 2
18.	Repeat interviews	Were repeat interviews carried out? If yes, how many?	Repeat interviews were not carried out, and have therefore not been reported
19.	Audio/visual recording	Did the research use audio or visual recording to collect the data?	Methods, Data preparation, page 4
20.	Field notes	Were field notes made during and/or after the interview or focus group?	Methods, Data preparation, page 4
21.	Duration	What was the duration of the interviews or focus group?	Methods, Data analysis, page 4
22.	Data saturation	Was data saturation discussed?	Methods, Data analysis, page 4
23.	Transcripts returned	Were transcripts returned to participants for comment and/or correction?	Methods, Data analysis, page 4
**Domain 3: analysis and findings**			
Data analysis			
24.	Number of data coders	How many data coders coded the data?	Methods, Data analysis, page 5
25.	Description of the coding tree	Did authors provide a description of the coding tree?	Methods, Data analysis, page 5
26.	Derivation of themes	Were themes identified in advance or derived from the data?	Methods, Data analysis, page 5
27.	Software	What software, if applicable, was used to manage the data?	Methods, Data analysis, page 5
28.	Participant checking	Did participants provide feedback on the findings?	No, but not specifically stated
Reporting			
29.	Quotations presented	Were participant quotations presented to illustrate the themes / findings? Was each quotation identified? *E.g. Participant number*	Results, Table 3
30.	Data and findings consistent	Was there consistency between the data presented and the findings?	Yes, demonstrated in the results/discussion, but not specifically stated
31.	Clarity of major themes	Were major themes clearly presented in the findings?	Results, page 7 et seq.
32.	Clarity of minor themes	Is there a description of diverse cases or discussion of minor themes?	Results, page 7 et seq.
