# Supplementary material for: Barriers and facilitators associated with the upscaling of the Transmural Trauma Care Model: a qualitative study
Source: BMC Health Serv Res. 2024 Feb 13;24:195. doi: 10.1186/s12913-024-10643-7 (PMC10865621; doi:10.1186/s12913-024-10643-7)
Supplement: Supplementary file 2 — Additional file 2. [file 12913_2024_10643_MOESM2_ESM.docx]

**Supplementary file 2**

***1.1 Topiclist trauma surgeon***

*Introduction*

*Personal questions*

- Age

- How long have you been working as a trauma surgeon?

- Where do you currently work all over?

- How long have you been working according to the TTCM model of care?

- What do you know about the TTCM care model?

If not familiar with the TTCM care model: Briefly explain.

*Open questions*

Question 1: What is your personal experience with the TTCM model of care?

- What is your role within the TTCM trial?

- What are your duties?

Question 2: How do you experience your working compared to before TTCM? (workload, quality of care for the patients)

- Which tasks are going well?

- Why are they going well (examples cite facilitators*)?

- What tasks are still a challenge/limitation (examples mention barriers*)?

- Can you elaborate on why ... is still a challenge?

- Why is this still a limitation?

- What do you think is the solution to this?

- How do you experience the cooperation with the hospital physical therapists?

- When can interprofessional collaboration lead to problems (explanation, examples)

- How do you experience cooperation with primary care physical therapists?

- How does this cooperation work in practice?

- Do you have confidence in the cooperation (why yes/no)?

- Tips/tops?

Question 3: In your opinion, should other hospitals in the Netherlands implement TTCM?

- What hindering and promoting factors do you expect in this regard?

- What problems do you expect/what problems could TTCM eliminate?

If you could give the model a score on a scale of 0-10, what would this be?

***1.2 Topiclist patients***

*Introduction*

- Thank you for taking the time for this interview. There will be no judgments and it is considered to be a neutral conversation.

- Brief explanation of TTCM and explanation of the purpose of the research and interview

- What are the hindering and promoting factors of the implementation of the TTCM, with respect to patients?

- Informed consent questions, emphasize anonymity

- Questions before we begin?

*Personal information*

- Age

- Gender

- What injury did you suffer and how did this happen?

- When did this happen?

- How long have you been treated using TTCM model?

- In which hospital are you being treated?

1. Experience:

- What is your personal experience with the TTCM model?

- How do you feel about the physical therapist at the consultation hour?

- Is he/she always present?

- Does he/she take a lot of initiative in the consultations (relative to the surgeon)?

2. How would you describe the cooperation in the consultation room between the trauma surgeon and the hospital based physical therapist?

- Were you referred to a physical therapist in your living area (from the network)?

- Did you experience the choice options you were given when being referred as a free choice?

- To the best of your knowledge, is there contact between the hospital and the physical therapy practice during your treatment?

- What was the transition from the hospital to the physical therapy practice like?

How do you experience the physical therapist's treatments?

- Does the advice you receive from the physical therapist outside the hospital match

with the advice given in the hospital?

- How is the treatment going so far?

3. Barriers:

Are there any negative factors that you have noticed during your treatment according to the

TTCM model?

- Why do you see this as a negative factor?

- Can you explain this in more detail?

- Where do you think this is due to? (Yourself, the physical therapist, the organization?)

Would you have liked this to be different? (If so, how? Maybe give examples)

4. Would insurance reimbursement be a factor for you to continue the pathway?

- For example, would you stop earlier than advised by the physical therapist?

- if your treatments were not reimbursed (anymore)?

Possible barriers: cooperation, materials, space, hours, time, contact researchers, location

Facilitators:

Are there any positive factors that you have noticed during your treatment according to the

TTCM model?

- Why do you see this as a positive factor?

- How has this helped you in your care process?

Possible facilitators: collaboration, materials, space, hours, time, contact researchers, location

Other question:

5. Do you have any other points you would like to make about the TTCM model?

6. What is your level of satisfaction, on a scale of 1 - 10?

***1.3 Topiclist hospital‐based physical therapists***

*Introduction*

*Personal questions*

- Age

- for how long working as a hospital based physiotherapist?

- For how long working with TTCM?

- Since when has TTCM been implemented in the hospital?

Question 1:

As a hospital based physical therapist, what is your personal experience with the TTCM which has recently been implemented in [name hospital]?

- Have you been directly involved in the implementation of the TTCM?

- What is your role regarding the TTCM which has recently been implemented in [name hospital]?

Question 2:

- What positive experiences have you had so far with the TTCM?

- Where do you think this is due to (the organization, yourself, the trauma surgeon or primary care physical therapist) Examples: Collaboration, resources, money, email traffic, researcher contact, location/space, patient contact, equivalency in post clinical checks/voice forward

Question 3:

- What negative experiences have you had so far the TTCM?

- Why do you see this as a negative experience?

- What do you think this is due to? (yourself, the trauma surgeon, primary care physical therapist, organization)

- What could you do to counter/diminish this negative experience? And what could be your role in this?

Question 4:

- Do you notice any difference in terms of quality of trauma care after implementation of the TTCM compared to regular care as before implementation?

- If so, what difference do you notice and to what extent do you think your new role as a second-line physical therapist in this model affects this?

Question 5:

- How do you find the collaboration going with the trauma surgeons?

- Is there equal cooperation?

- How is the communication with the trauma surgeons?

- Did you ever have contact with trauma surgeons prior to implementation?

- If so, how often and how was that contact?

Question 6:

- How do you find the collaboration going with the primary care physical therapists?

- How is the communication going?

- Is email traffic about the patient going smoothly?

- What tool is used for your communication with the primary care physical therapists?

- Are the emails you receive back from the primary care network clear?

- Is a standard format used?

Question 7:

- How do you feel the contact as a second-line physical therapist goes with the patients within the TTCM ?

- Do you have enough contact with the patient?

- Does advice arrive well and clearly with the patient?

Question 8:

- How do you feel the post clinical checkups are done together with the trauma surgeon and patient?

- How often are those checkups scheduled?

- Do you find that your advice can help both the trauma surgeon and patient?

- Regarding the establishment of individual treatment goals, how does this involve collaboration with the trauma surgeon and patient?

- Are treatment recommendations drawn up together with the trauma surgeon and do you complement each other in this?

Question 9:

- In your role as a hospital based physical therapist in the TTCM, you advise the patient but you also have an important communicative role to the primary care network. Where do you feel your strengths lie most, on the outpatient clinic or towards the network?

- As you know, the TTCM consists of four major components ((1) A joint outpatient consultations by a multidisciplinary team consisting of a trauma surgeon and a hospital‐based physiotherapist (HBP); 2) Coordination and individual goal-setting; 3) A network of specialized network physiotherapists (NPs); and 4) Secured email traffic between HBPs and NPs.))

- Which component do you think is most relevant and where do you think the most growth lies?

Question 10:

- What would you recommend in terms of further scaling up TTCM in the rest of the hospitals in the Netherlands?

- Do you think that other hospitals can also start to benefit from it and that the TTCM should therefore be expanded?

- If the TTCM is going to be implemented in multiple hospitals, what would you advise second-line physical therapists to make the implementation of the TTCM as successful as possible?

Closing questions:

- Do you have anything to add to the interview, or important points I forgot to ask?

***1.4 Topiclist specialized primary care physiotherapists***

*Introduction*

*Personal questions*

- Age

- For how long working as a hospital based physiotherapist?

- For how long working with TTCM?

How do you experience working according to the TTCM?

- What is your personal experience with the TTCM model?- Wat zijn uw taken binnen het TTCM-model?

Barriers:

- What disadvantages do you experience while working according to the trauma rehabilitation network?

- What makes these factors limiting?

- What do you think would be a good solution for these barriers?

- Cooperation, resources, materials, space, money, hours, time, training, contact researchers, location

Facilitators:

- What advantages do you experience while working according to the trauma rehabilitation network?

- What makes these factors conducive?

- Cooperation, resources, materials, space, money, hours, time, training, contact researchers, location

Additional questions:

- How do you experience the cooperation with the hospital based physical therapists?

- How do you experience the cooperation with the trauma surgeons?

- How do you experience the quality of care for the patients?

- How do you experience the workload of the TTCM project?

- Is it increasing or decreasing?

- Do you still have enough time for other patients?

- Do you have to pay dues to participate in the trauma rehabilitation network?

- How do you experience the mental aspect of trauma care in the trauma rehabilitation network?
