# Supplementary material for: Barriers and facilitators associated with the upscaling of the Transmural Trauma Care Model: a qualitative study
Source: BMC Health Serv Res. 2024 Feb 13;24:195. doi: 10.1186/s12913-024-10643-7 (PMC10865621; doi:10.1186/s12913-024-10643-7)
Supplement: Supplementary file 3 — Additional file 3. [file 12913_2024_10643_MOESM3_ESM.docx]

**Supplementary file 3** Constellation approach

This approach assumes that a healthcare system consists of so-called constellations, i.e. a set of interrelated practices and relevant, interrelated, structuring elements that define and fulfill a function in the more extensive system (22). To meet their diverse needs, healthcare systems consist of many nested complementing and competing constellations and (sub)constellations (58). Within a constellation, there is a continuous interaction between the three elements of the ‘structure, culture and practice triplet’ (22). ‘Structure’ consists of the physical structures and resources, enforced regulations and legal rights, economic resources, and other material elements that structure behavior within a constellation (e.g. compatibility of electronic patient records). ‘Culture’ refers to the paradigms, norms and values, and other immaterial elements that structure behavior in practices (e.g. the willingness of different departments working together at the outpatient clinic). ‘Practice’ involves the typical operational routines which the actors within the constellation undertake. Actors are the individuals (e.g., patients, physicians, managers) or groups (e.g., insurance companies, departments) who work or act in a particular constellation. For the TTCM, several nested constellations can be recognized, for example, the outpatient clinic for trauma patients on the one hand and the primary/tertiary care network practices on the other hand. Moreover, both the hospital and the primary/tertiary care network practices are part of a bigger constellation in which insurers and policymakers act in a particular structure and culture. Dynamics, such as those created by the upscaling of the TTCM, provide an opportunity for change. When the change process leads to a fundamental shift in structure, culture, and practice, a transition of the constellation has occurred. The driving force of change is the sense of urgency for change by ‘key actors’ within a constellation (59). These actors initiate and push for change on the structural, cultural, and practical levels (60). To achieve a transition, the relevant actors need to develop a collective sense of urgency to change and develop new competencies (knowledge, attitude, and skills). Scaling up involves implementing the results of niche experiments in the existing structure, culture, and practice (18, 59, 61).
